# Supplementary material for: Who cares about mental health? Benchmarking the issue importance of mental health for American voters”
Source: PLoS One. 2026 Mar 18;21(3):e0342486. doi: 10.1371/journal.pone.0342486 (PMC12998877; doi:10.1371/journal.pone.0342486)
Supplement: S1 Appendix — (DOCX) [file pone.0342486.s001.docx]

**S1 Appendix. Questionnaire.**

*This appendix displays the text and instructions for the module questions, including the wording of Common Content items used in the experiment. The wording of the remaining Common Content items is publicly accessible in the CES guides and searchable on the CES website:* [*https://cooperativeelectionstudy.shinyapps.io/ccsearch/*](https://cooperativeelectionstudy.shinyapps.io/ccsearch/)

300

SINGLE CHOICE

Mental health legislation support

Do you support or oppose the following proposal?

Change health insurance rules and reimbursement rates to improve access to mental health care for all, including lower-income people, children, and the elderly.

1  Support

2  Oppose

301a

Mental health legislation strong or weak support

*Only display this question for those who answered 1 “Support” to 300.*

Which of these best describes your support for the proposal you just saw?

1. Strong support
2. Weak support

301b

Mental health legislation strong or weak opposition

*Only display this question for those who answered 2 “Oppose” to 300.*

Which of these best describes your opposition to the proposal you just saw?

1. Strong opposition
2. Weak opposition

311, 312, 313, 314, 315, 316

SINGLE CHOICE / CONJOINT EXPERIMENT

Issue importance conjoint questions

*Conjoint experiment – for each question, please randomly vary the policy issues on each row from the list of proposals provided, and randomly display “supports” or “opposes” in each cell (candidates may have the same position on one or both displayed issues). Policy issues should be randomly selected without replacement for each table/question (i.e., no table should show the same issue on both rows, but respondents may see the same policy issue more than once in separate tables). Please note that the wording for most of these is identical to items from the draft pre-election Common Content questionnaire.*

*List of issues:*

- *Increase the number of border patrols on the US-Mexican border. [Same as CC24_323b]*
- *Expand access to abortion, including making it more affordable, broadening the types of providers who can offer care, and protecting access to abortion clinics. [same as CC24_324d]*
- *Give the Environmental Protection Agency power to regulate carbon dioxide emissions [same as CC24_326a]*
- *Expand federal tax incentives to encourage developers to build homes for people who make less than half of the average income in your area [same as CC24_328b]*
- *Repeal the Affordable Care Act [same as CC24_328d]*
- *Forgive up to $20,000 of student loan debt for each person [same as CC24_328f]*
- *Spend $150 billion a year for 8 years on construction and repair of roads and bridges, rail, public transit, airports, water systems, broadband internet, and electric grid [same as CC24_341d]*
- *Allow tax rates on those earning $400,000 or more a year to rise to 35 percent [CC24_341c]*
- *Ban TikTok unless China sells it to a US company [same as CC24_340d]*
- *Change health insurance rules and reimbursement rates to improve access to mental health care for all, including lower-income people, children, and the elderly [same as 300]*

Imagine you had to choose in the upcoming election between two candidates for Congress, A and B. The two candidates were asked about their position on two proposals. Their answers are shown below.

| Issue | Candidate A | Candidate B |
| --- | --- | --- |
| [Proposal 1] | Supports/opposes the proposal | Supports/opposes the proposal |
| [Proposal 2] | Supports/opposes the proposal | Supports/opposes the proposal |

Given only the above information, for which candidate would you vote? If the positions are the same, please pick either one.

1. Candidate A
2. Candidate B

*Example:*

*Imagine you had to choose in the upcoming election between two candidates for Congress, A and B. The two candidates were asked about their position on two proposals. Their answers are shown below.*

| *Issue* | *Candidate A* | *Candidate B* |
| --- | --- | --- |
| *Change health insurance rules and reimbursement rates to improve access to mental health care for all, including lower-income people, children, and the elderly* | *Supports the proposal* | *Opposes the proposal* |
| *Expand access to abortion, including making it more affordable, broadening the types of providers who can offer care, and protecting access to abortion clinics.* | *Opposes the proposal* | *Opposes the proposal* |

*Given only the above information, for which candidate would you vote? If the positions are the same, please pick either one.*

1. *Candidate A*
2. *Candidate B*
