# Supplementary material for: Who cares about mental health? Benchmarking the issue importance of mental health for American voters”
Source: PLoS One. 2026 Mar 18;21(3):e0342486. doi: 10.1371/journal.pone.0342486 (PMC12998877; doi:10.1371/journal.pone.0342486)
Supplement: S2 Appendix — (DOCX) [file pone.0342486.s002.docx]

**S2 Appendix. Descriptive Statistics.**

**Table S2.1. Distributions of moderating variables from the Common Content.**

|  | Summary |
| --- | --- |
| N | 1,000 |
| Self-reported health |  |
| Fair/Poor | 25.2% |
| Good | 38.0% |
| Very good | 27.7% |
| Excellent | 9.1% |
| Health insurance type |  |
| Employer | 39.8% |
| Gov't | 41.7% |
| Self | 8.2% |
| None | 10.3% |
| Party ID |  |
| Democrat | 33.8% |
| Independent | 38.6% |
| Republican | 27.6% |
| Ideology |  |
| Liberal | 28.4% |
| Moderate | 40.6% |
| Conservative | 31.0% |
| Education |  |
| HS/less | 35.5% |
| Some coll. | 18.5% |
| 2yr | 12.6% |
| 4yr/postgrad | 33.4% |
| Family income tercile |  |
| <$40k | 35.5% |
| $40k-<$100k | 37.3% |
| $100k+ | 27.2% |
| Age |  |
| 18 - 44 | 45.0% |
| 45 - 63 | 29.2% |
| 64 - 93 | 25.8% |
| Man |  |
| No | 52.3% |
| Yes | 47.7% |
| Race/ethnicity |  |
| White | 65.4% |
| Black | 12.3% |
| Hispanic | 10.6% |
| All others | 11.7% |

Table displays weighted percentages, which may not add to 100 due to rounding. Percentages are calculated at the respondent level.

**Table S2.2. Proportion of respondents supporting each policy proposal.**

|  | Summary |
| --- | --- |
| N | 1,000 |
| Respondent supports: Mental health | 91% |
| Respondent supports: Border security | 74% |
| Respondent supports: Abortion access | 60% |
| Respondent supports: Carbon emissions | 62% |
| Respondent supports: Affordable housing | 73% |
| Respondent supports: ACA repeal | 38% |
| Respondent supports: Student debt forgiveness | 54% |
| Respondent supports: Infrastructure spending | 82% |
| Respondent supports: Billionaire tax | 68% |
| Respondent supports: Banning TikTok | 53% |

Table displays weighted percentage of respondents selecting “support.”
