# Supplementary material for: Who cares about mental health? Benchmarking the issue importance of mental health for American voters”
Source: PLoS One. 2026 Mar 18;21(3):e0342486. doi: 10.1371/journal.pone.0342486 (PMC12998877; doi:10.1371/journal.pone.0342486)
Supplement: S3 Appendix — (DOCX) [file pone.0342486.s003.docx]

**S3 Appendix. Conjoint Methodology.**

This study adapts and applies the methodology laid out in Alvarez and Morrier 2025 for assessing issue importance using conjoint experiments. The main text describes the design of the conjoint experiments, while this appendix provides more detail on the methodological approach to analyzing the data.

Alvarez and Morrier develop two versions of the average marginal component effect (AMCE) for issue $\mathcal{l}$: $\Delta_{\mathcal{l}}$ and $\tilde{\Delta}_{\mathcal{l}}$. The latter incorporates respondent expressions of neutrality on policy issues. This is not possible in the present study due to the strictly dichotomous nature of the CES Common Content policy questions. Thus, I estimate the equivalent of $\Delta_{\mathcal{l}}$, which Alvarez and Morrier summarize with this formula:

$$\Delta_{\mathcal{l}}\mathbb{=P}\left( Voting for a candidate \right| Agreeing with the candidate on issue\mathcal{l)-}\mathbb{P}\left( Voting for a candidate \right| Disagreeing with the candidate on issue\mathcal{l)}$$

Each conjoint table contains three other pieces of substantive information (aside from the candidate’s position on issue $\mathcal{l}$) that are likely to affect respondent’s reported vote choice: the candidate’s position on the other issue ($\mathcal{-l}$), and the other candidate’s positions on both $\mathcal{l}$ and $-\mathcal{l}$. Importantly, the randomization of both issues and all four candidate positions ensures that none of the three other positions will systematically bias $\Delta_{\mathcal{l}}$, which “averages out” these other factors.

While $\Delta_{\mathcal{l}}$ can be estimated with a simple test for a difference in means or proportions, the candidate order effect discussed in the main text (the systematic preference for the randomly generated “candidate A” over “candidate B”) introduces statistical noise. Using multivariate regression (linear probability model) to control for candidate table position helps to substantially increase precision. The regression formula for the agreement effect (AMCE) of each issue $\mathcal{l}$ as displayed in Fig 1 is:

$$Y_{j}=\beta_{0}+\beta_{1}\theta_{\mathcal{l}j}+\beta_{2}B_{j}+\epsilon$$

Where $Y_{j}$ is an indicator of choosing to vote for candidate $j$ over the other candidate, $\theta_{\mathcal{l}j}$ is an indicator of agreement (1) or disagreement (0) with candidate $j$ on issue $\mathcal{l}$, $B_{j}$ is an indicator of whether candidate $j$appears in the B (second) column of the table, and $\epsilon$ is the random error term. $\beta_{1}$, the coefficient for $\theta_{\mathcal{l}j}$, is the estimate of the AMCE for issue $\mathcal{l}$.

The heterogeneous effects analysis (results shown in Fig 2) modifies this model to incorporate indicators of respondent demographic and political categories and their interactions with $\theta_{\mathcal{l}j}$. For simplicity, assume a dichotomous measure of a respondent characteristic that can be measured with a single indicator $X$:

$$Y_{j}=\beta_{0}+\beta_{1}\theta_{\mathcal{l}j}+\beta_{2}B_{j}+\beta_{3}X+\beta_{4}\theta_{\mathcal{l}j}\times X+\epsilon$$

The estimates of the conditional agreement effects (AMCE) for issue $\mathcal{l}$ are calculated with $\beta_{1}+\beta_{4}X$. For the group for which $X=0$ the conditional AMCE is $\beta_{1}+\beta_{4}0=$ $\beta_{1}$, while that for the group for which $X=1$ is $\beta_{1}+\beta_{4}1=\beta_{1}+\beta_{4}$. For respondent variables with more than two categories, this approach is modified with additional indicator variables and their interactions with $\theta_{\mathcal{l}j}$.

For all regressions, I use the module sampling weights provided by YouGov and robust standard errors clustered by respondent.
