# Supplementary material for: Who cares about mental health? Benchmarking the issue importance of mental health for American voters”
Source: PLoS One. 2026 Mar 18;21(3):e0342486. doi: 10.1371/journal.pone.0342486 (PMC12998877; doi:10.1371/journal.pone.0342486)
Supplement: S4 Appendix — (DOCX) [file pone.0342486.s004.docx]

**S4 Appendix. Full Regression Results.**

**Table S4.1. Full regression results for conjoint analyses displayed in Figure 1.**

|  | **Mental health** | **Border security** | **Abortion access** | **Carbon emissions** | **Affordable housing** |
| --- | --- | --- | --- | --- | --- |
| Agreement | 0.273 (0.027) | 0.226 (0.029) | 0.300 (0.029) | 0.179 (0.032) | 0.206 (0.031) |
| Table position | -0.098 (0.041) | -0.160 (0.042) | -0.156 (0.042) | -0.088 (0.044) | -0.115 (0.043) |
| Constant | 0.505 (0.063) | 0.626 (0.065) | 0.590 (0.067) | 0.547 (0.070) | 0.571 (0.067) |
|  |  |  |  |  |  |
| R-squared | 0.085 | 0.078 | 0.114 | 0.038 | 0.057 |
| F | 55.35 | 36.58 | 69.84 | 20.48 | 25.97 |
| N | 2422 | 2452 | 2420 | 2350 | 2378 |
|  |  |  |  |  |  |
|  | **ACA repeal** | **Student debt forgiveness** | **Infrastructure spending** | **Billionaire tax** | **Banning TikTok** |
| Agreement | 0.266 (0.035) | 0.281 (0.028) | 0.266 (0.030) | 0.256 (0.028) | 0.207 (0.028) |
| Table position | -0.145 (0.043) | -0.169 (0.041) | -0.142 (0.043) | -0.063 (0.042) | -0.084 (0.045) |
| Constant | 0.583 (0.069) | 0.619 (0.066) | 0.585 (0.068) | 0.461 (0.064) | 0.521 (0.072) |
|  |  |  |  |  |  |
| R-squared | 0.091 | 0.106 | 0.090 | 0.069 | 0.051 |
| F | 38.43 | 73.30 | 48.57 | 42.23 | 34.08 |
| N | 2444 | 2320 | 2326 | 2452 | 2436 |

Survey weights used. Standard errors clustered by respondent displayed in parentheses. See Appendix S2 for descriptive statistics for these variables.

**Table S4.2. Full regression results for heterogeneous effects analyses displayed in Figure 2 for self-reported health and health insurance status.**

|  | **(1)** | **(2)** |
| --- | --- | --- |
|  | **Self-reported health** | **Insurance status** |
|  |  |  |
| Agree | 0.305 (0.068) | 0.288 (0.039) |
|  |  |  |
| Table position | -0.097 (0.040) | -0.094 (0.041) |
|  |  |  |
| Good | 0.016 (0.042) |  |
|  |  |  |
| Very good | 0.055 (0.044) |  |
|  |  |  |
| Excellent | 0.118 (0.057) |  |
|  |  |  |
| Agree X Good | 0.008 (0.077) |  |
|  |  |  |
| Agree X Very good | -0.078 (0.081) |  |
|  |  |  |
| Agree X Excellent | -0.215 (0.099) |  |
|  |  |  |
| Gov't |  | 0.022 (0.030) |
|  |  |  |
| Self |  | -0.081 (0.069) |
|  |  |  |
| None |  | 0.033 (0.069) |
|  |  |  |
| Agree X Gov't |  | -0.028 (0.058) |
|  |  |  |
| Agree X Self |  | 0.064 (0.102) |
|  |  |  |
| Agree X None |  | -0.072 (0.126) |
|  |  |  |
| Constant | 0.475 (0.072) | 0.490 (0.065) |
|  |  |  |
| Observations | 2422 | 2388 |
| R-squared | 0.089 | 0.083 |
| F | 16.285 | 15.019 |

Survey weights used. Standard errors clustered by respondent displayed in parentheses. Excluded categories: Fair/poor (1), employer (2). See Appendix S2 for descriptive statistics for these variables.

**Table S4.3. Full regression results for heterogeneous effects analyses displayed in Figure 2 for party identification and ideology.**

|  | **(1)** | **(2)** |
| --- | --- | --- |
|  | **Party identification** | **Ideology** |
|  |  |  |
| Agree | 0.296 (0.051) | 0.357 (0.047) |
|  |  |  |
| Table position | -0.098 (0.041) | -0.097 (0.041) |
|  |  |  |
| Independent | 0.023 (0.036) |  |
|  |  |  |
| Republican | 0.021 (0.038) |  |
|  |  |  |
| Agree X Independent | -0.042 (0.066) |  |
|  |  |  |
| Agree X Republican | -0.027 (0.071) |  |
|  |  |  |
| Moderate |  | 0.056 (0.035) |
|  |  |  |
| Conservative |  | 0.076 (0.033) |
|  |  |  |
| Agree X Moderate |  | -0.099 (0.067) |
|  |  |  |
| Agree X Conservative |  | -0.146 (0.061) |
|  |  |  |
| Constant | 0.490 (0.067) | 0.458 (0.068) |
|  |  |  |
| Observations | 2422 | 2422 |
| R-squared | 0.085 | 0.088 |
| F | 19.110 | 21.599 |

Survey weights used. Standard errors clustered by respondent displayed in parentheses. Excluded categories: Democrat (1), liberal (2). See Appendix S2 for descriptive statistics for these variables.

**Table S4.4. Full regression results for heterogeneous effects analyses displayed in Figure 2 for education and family income.**

|  | **(1)** | **(2)** |
| --- | --- | --- |
|  | **Education** | **Family income** |
|  |  |  |
| Agree | 0.240 (0.051) | 0.196 (0.052) |
|  |  |  |
| Table position | -0.098 (0.041) | -0.074 (0.042) |
|  |  |  |
| Some coll. | -0.038 (0.043) |  |
|  |  |  |
| 2yr | 0.017 (0.041) |  |
|  |  |  |
| 4yr/postgrad | -0.038 (0.035) |  |
|  |  |  |
| Agree X Some coll. | 0.093 (0.076) |  |
|  |  |  |
| Agree X 2yr | -0.004 (0.085) |  |
|  |  |  |
| Agree X 4yr/postgrad | 0.057 (0.066) |  |
|  |  |  |
| $40k-<$100k |  | -0.051 (0.033) |
|  |  |  |
| $100k+ |  | -0.063 (0.038) |
|  |  |  |
| Agree X $40k-<$100k |  | 0.097 (0.064) |
|  |  |  |
| Agree X $100k+ |  | 0.122 (0.074) |
|  |  |  |
| Constant | 0.521 (0.069) | 0.510 (0.068) |
|  |  |  |
| Observations | 2422 | 2268 |
| R-squared | 0.087 | 0.080 |
| F | 16.818 | 19.040 |

Survey weights used. Standard errors clustered by respondent displayed in parentheses. Excluded categories: HS/less (1), <$40k (2). See Appendix S2 for descriptive statistics for these variables.

**Table S4.5. Full regression results for heterogeneous effects analyses displayed in Figure 2 for age, gender, and race/ethnicity.**

|  | **(1)** | **(2)** | **(3)** |
| --- | --- | --- | --- |
|  | **Age** | **Gender (man)** | **Race/ethnicity** |
|  |  |  |  |
| Agree | 0.261 (0.041) | 0.289 (0.036) | 0.300 (0.031) |
|  |  |  |  |
| Table position | -0.098 (0.041) | -0.097 (0.040) | -0.097 (0.041) |
|  |  |  |  |
| 45 - 63 | -0.022 (0.035) |  |  |
|  |  |  |  |
| 64 - 93 | 0.016 (0.031) |  |  |
|  |  |  |  |
| Agree X 45 - 63 | 0.055 (0.069) |  |  |
|  |  |  |  |
| Agree X 64 - 93 | -0.018 (0.057) |  |  |
|  |  |  |  |
| Yes |  | 0.016 (0.028) |  |
|  |  |  |  |
| Agree X Yes |  | -0.033 (0.054) |  |
|  |  |  |  |
| Black |  |  | 0.066 (0.055) |
|  |  |  |  |
| Hispanic |  |  | 0.023 (0.058) |
|  |  |  |  |
| All others |  |  | 0.021 (0.041) |
|  |  |  |  |
| Agree X Black |  |  | -0.127 (0.108) |
|  |  |  |  |
| Agree X Hispanic |  |  | -0.045 (0.100) |
|  |  |  |  |
| Agree X All others |  |  | -0.060 (0.073) |
|  |  |  |  |
| Constant | 0.508 (0.066) | 0.496 (0.062) | 0.490 (0.064) |
|  |  |  |  |
| Observations | 2422 | 2422 | 2422 |
| R-squared | 0.086 | 0.085 | 0.087 |
| F | 20.593 | 27.768 | 16.087 |

Survey weights used. Standard errors clustered by respondent displayed in parentheses. Excluded categories: 18-44 (1), No (not a man) (2), White (3). See Appendix S2 for descriptive statistics for these variables.

**Table S4.6. Full regression results for heterogeneous effects analyses for respondent mental health issue position.**

|  | **(1)** | **(2)** |
| --- | --- | --- |
|  | **Strong vs weak** | **Ordinal** |
|  |  |  |
| Agree | 0.135 (0.058) | 0.156 (0.108) |
|  |  |  |
| Table position | -0.095 (0.041) | -0.095 (0.041) |
|  |  |  |
| Strong support/opposition | -0.099 (0.033) |  |
|  |  |  |
| Agree X Strong support/opposition | 0.179 (0.065) |  |
|  |  |  |
| Weak opposition |  | 0.014 (0.092) |
|  |  |  |
| Weak support |  | 0.048 (0.084) |
|  |  |  |
| Strong support |  | -0.059 (0.079) |
|  |  |  |
| Agree X Weak opposition |  | 0.037 (0.152) |
|  |  |  |
| Agree X Weak support |  | -0.036 (0.127) |
|  |  |  |
| Agree X Strong support |  | 0.164 (0.113) |
|  |  |  |
| Constant | 0.574 (0.068) | 0.533 (0.097) |
|  |  |  |
| Observations | 2408 | 2408 |
| R-squared | 0.091 | 0.092 |
| F | 33.041 | 16.749 |

Survey weights used. Standard errors clustered by respondent displayed in parentheses. Excluded categories: weak support/opposition (1), strong opposition (2). See Appendix S2 for descriptive statistics for these variables.
