# Supplementary material for: Who cares about mental health? Benchmarking the issue importance of mental health for American voters”
Source: PLoS One. 2026 Mar 18;21(3):e0342486. doi: 10.1371/journal.pone.0342486 (PMC12998877; doi:10.1371/journal.pone.0342486)
Supplement: Attachment — Submitted filename: PONE-D-25-40424_comments.docx [file pone.0342486.s005.docx]

Comment: Introduction:

Could you add more about the rationale? And a clear objective. Your objective has been spread all over the introduction. I understand what you intend to do, however, we need a clear and specific written objective.

Comment: Data and Variables : you said you sampled 1000 randomly, but how many where there in total? And did you do the randomization? If you didn’t opt for any randomization technique, then you should probably say that you sampled them using convenience sampling. Otherwise, explicitly mention how you randomized them.

Comment: in line 163-165, you said “implement the conjoint experiment using a combination of 164 original questions and items from the CES Common Content 165 questionnaire.”. Since you are referring to something, putting a reference would be a better choice.

Comment: In line 209-211, you said, “I re-code the randomized candidate issue positions to 210 indicate whether the candidate agrees with the respondent (1) or 211 not (0). ”. What do you mean by re-code? Clarify a little bit. Did you mean post-coding?

Comment: In line 211-212, you said “These agreement variables are the key independent 212 variables (see “Analysis” for more on methodology)”, here you are referring to something you described later. Usually reference is made to something described earlier. However, a little more is required to understand the concept of “agreement variables”. You didn’t mention this terminology earlier.

Comment: Then in line 213-216, you said “For the heterogeneous effects analysis, I make use of the 214 Common Content’s battery of demographic and political questions, 215 collapsing some categories to achieve adequate subgroup sample 216 sizes.”. Reference is needed here to justify that whatever you are doing scientifically appropriate.

Comment: The authors should indicate whether the study adhered to established ethical standards (e.g., the Declaration of Helsinki). Although the manuscript states that the study was approved by the Institutional Review Board (IRB), it would be helpful to explicitly mention compliance with relevant ethical guidelines. If the research complied with these standards, please state this clearly in the Ethics section.

Comment: In line 236-237, you mentioned “I follow the analytical strategy of Alvarez and Morrier with a 237 slight modification. ”. Again you are referring to something, need to put a reference.

Comment: Do you have explicit reason behind using 90% CI. Usually 95% is used. If you are using 90%, you made concrete justification for that.

Comment: In the discussion and conclusion you mention relatively small sample size as one of you limitations. However, 1000 participant is not a small sample.

Comment: Appendix S2. The frequency table is not representative. If you could add percentage along with the frequency, it would better represent.

Comment: Appendix S4 is a bit confusing. Try to incorporate more information about the items of table in the suffix.
