# Supplementary material for: Who cares about mental health? Benchmarking the issue importance of mental health for American voters”
Source: PLoS One. 2026 Mar 18;21(3):e0342486. doi: 10.1371/journal.pone.0342486 (PMC12998877; doi:10.1371/journal.pone.0342486)
Supplement: Attachment — Submitted filename: MH Issue Importance revision letter.docx [file pone.0342486.s007.docx]

Dear editor and reviewers,

Thank you for taking the time to review my manuscript (“Who cares about mental health? Benchmarking the issue importance of mental health for American voters,” PONE-D-25-40424) and for the opportunity to submit this revision addressing your comments. I hope that this revision is responsive to all of your insightful and helpful comments and questions.

This letter enumerates the changes I have made in response to your comments (reproduced here in italics). I assume that reviewers will prefer to view the version of the revision with tracked changes, so all of the line number references in this letter refer to that version, not the version with tracked changes removed.

**Journal Requirements**

*When submitting your revision, we need you to address these additional requirements.*

*1. Please ensure that your manuscript meets PLOS ONE's style requirements, including those for file naming. The PLOS ONE style templates can be found at*

[*https://journals.plos.org/plosone/s/file?id=wjVg/PLOSOne_formatting_sample_main_body.pdf*](https://journals.plos.org/plosone/s/file?id=wjVg/PLOSOne_formatting_sample_main_body.pdf) *and*

[*https://journals.plos.org/plosone/s/file?id=ba62/PLOSOne_formatting_sample_title_authors_affiliations.pdf*](https://nam02.safelinks.protection.outlook.com/?url=https%3A%2F%2Ftrack.editorialmanager.com%2FCL0%2Fhttps%3A%252F%252Fjournals.plos.org%252Fplosone%252Fs%252Ffile%253Fid%3Dba62%252FPLOSOne_formatting_sample_title_authors_affiliations.pdf%2F1%2F010f019b011b8e3a-a311673f-f891-4469-bd97-5a5928a53bb0-000000%2FTtn8d306MZbCCDG2RAnAXSCNSPQS_oZthutRjW--DFs%3D240&data=05%7C02%7Chaselswerdtj%40missouri.edu%7C501785a1bbe74df2376208de36d13c84%7Ce3fefdbef7e9401ba51a355e01b05a89%7C0%7C0%7C639008469798595547%7CUnknown%7CTWFpbGZsb3d8eyJFbXB0eU1hcGkiOnRydWUsIlYiOiIwLjAuMDAwMCIsIlAiOiJXaW4zMiIsIkFOIjoiTWFpbCIsIldUIjoyfQ%3D%3D%7C0%7C%7C%7C&sdata=x7qQVQyToZoHMWP7lCxuhqTRmQP0LWXyfwBD%2FO5BJ9E%3D&reserved=0)

I have changed the formatting of the title page, headings, references, and figures (including file type and names) to match these examples.

*2. Thank you for stating the following financial disclosure:*

*“The survey module was paid for with internal funding from the Truman School of Government & Public Affairs at the University of Missouri.”*

*Please state what role the funders took in the study.  If the funders had no role, please state: "The funders had no role in study design, data collection and analysis, decision to publish, or preparation of the manuscript."*

*If this statement is not correct you must amend it as needed.*

*Please include this amended Role of Funder statement in your cover letter; we will change the online submission form on your behalf.*

I have added this text to the revised funding statement included in the cover letter.

*3. Thank you for stating the following in the Acknowledgments Section of your manuscript:*

*“The author also acknowledges the Truman School of Government and Public Affairs for funding the CES module.”*

*We note that you have provided funding information that is not currently declared in your Funding Statement. However, funding information should not appear in the Acknowledgments section or other areas of your manuscript. We will only publish funding information present in the Funding Statement section of the online submission form.*

*Please remove any funding-related text from the manuscript and let us know how you would like to update your Funding Statement. Currently, your Funding Statement reads as follows:*

*“The survey module was paid for with internal funding from the Truman School of Government & Public Affairs at the University of Missouri.”*

*Please include your amended statements within your cover letter; we will change the online submission form on your behalf.*

I have removed the funding-related text from the “Acknowledgments” section, and submitted a revised version of the funding statement in the cover letter (see above).

*4. Please note that your Data Availability Statement is currently missing the DOI/accession number of each dataset OR a direct link to access each database. If your manuscript is accepted for publication, you will be asked to provide these details on a very short timeline. We therefore suggest that you provide this information now, though we will not hold up the peer review process if you are unable.*

I have posted the data and replication code and added the DOI to the Data Availability Statement.

*5. Please include your full ethics statement in the ‘Methods’ section of your manuscript file. In your statement, please include the full name of the IRB or ethics committee who approved or waived your study, as well as whether or not you obtained informed written or verbal consent. If consent was waived for your study, please include this information in your statement as well.*

I have edited the existing text in the Research Design, Data and Methods section so that all of this information appears in one place and the full name of the IRB is displayed with no abbreviation – see lines 254-259 in the tracked-changes revision.

*6. If the reviewer comments include a recommendation to cite specific previously published works, please review and evaluate these publications to determine whether they are relevant and should be cited. There is no requirement to cite these works unless the editor has indicated otherwise.*

Thank you for this guidance.

*7. Please review your reference list to ensure that it is complete and correct. If you have cited papers that have been retracted, please include the rationale for doing so in the manuscript text, or remove these references and replace them with relevant current references. Any changes to the reference list should be mentioned in the rebuttal letter that accompanies your revised manuscript. If you need to cite a retracted article, indicate the article’s retracted status in the References list and also include a citation and full reference for the retraction notice.*

The updated reference list is complete and correct, and does not cite any retracted papers. I have added citations to the following works that were not included in the original manuscript:

Bernardi L. Mental Health and Political Representation: A Roadmap. Front Polit Sci. 2021 Jan 29;2:587588.

Fournier P, Blais A, Nadeau R, Gidengil E, Nevitte N. Issue Importance and Performance Voting. Political Behavior. 2003 Mar 1;25(1):51–67.

Gallup Poll Social Series: Environment Final Topline [Internet]. Gallup News Service; 2025 Mar [cited 2026 Jan 12]. Available from: <https://news.gallup.com/file/poll/659009/2025_04_09%20Energy%20Topline%20and%20Tabs_MIP.pdf>

Krosnick JA. The role of attitude importance in social evaluation: a study of policy preferences, presidential candidate evaluations, and voting behavior. J Pers Soc Psychol. 1988 Aug;55(2):196–210.

Magni G, Reynolds A. Voter attitudes and politicians with health conditions and disabilities. Social Science Quarterly. 2024;105(6):1923–37.

Moniz PM, Wlezien CW. Issue Salience and Political Decisions. In: The Oxford Encyclopedia of Political Decision Making [Internet]. Oxford University Press; 2021 [cited 2024 June 20]. Available from: <https://www.oxfordreference.com/display/10.1093/acref/9780190622848.001.0001/acref-9780190622848-e-1361>

Nelson TE, Oxley ZM. Issue Framing Effects on Belief Importance and Opinion. The Journal of Politics. 1999 Nov;61(4):1040–67.

Schuessler J, Freitag M. Power Analysis for Conjoint Experiments [Internet]. SocArXiv; 2020 [cited 2025 Aug 11]. Available from: <https://osf.io/9yuhp>

Stokes D. Valence Politics. In: Kavanagh D, editor. Electoral Politics. Oxford University Press Oxford; 1992. p. 141–64.

Tourangeau R, Couper MP, Conrad FG. “Up Means Good.” Public Opin Q. 2013;77(Suppl 1):69–88.

**Additional Editor Comments** *Kindly take into account in your revised version all the comments raised by the reviewers and be keen to provide answers, refutations or suitable amendment in the manuscript. I would like the authors also to be aware about recent works related to voting behavior of American voters (*[*https://doi.org/10.1142/S0129183124501900*](https://doi.org/10.1142/S0129183124501900)*,* [*https://doi.org/10.1371/journal.pone.0331959*](https://doi.org/10.1371/journal.pone.0331959)*) that may be relevant to their work, if they find informative and suitable.*

I thank the editor for bringing these works to my attention. While both are interesting, they do not deal with the role of policy attitudes in vote choice, which is the focus of this study.

*The manuscript submitted is of interesting and original value, but needs to be revised according to all comments raised in the revision process.*

**Reviewer #1**

*Thank you for the opportunity to review this very interesting manuscript on public opinion towards mental health in the US and its implications for candidate support. Overall, I find the paper original and I believe it makes a nice contribution to the mental health and politics subfield. Below I have some comments for the author which I hope will help improve the paper.

It is interesting that mental health seems to be more important than other issues in 2024; although I would distinguish between support for and salience of the issue. I think the author can dig deeper here. When polling companies in the US like Gallup ask about the most important issue/problem (MII/MIP) in the country through an open-ended question, has mental health ever come up as one of the issues? Certainly, it was not the case in the past, but I wonder whether this has changed given that people have become more aware of the issue. If the author finds such evidence, then this can help with the external validity of the research. If the author, instead, does not find such evidence, then it is worth discussing the point anyway because when people are primed, they do think about mental health, as the paper shows. This is still a relevant point when thinking about priming effects and what polls should ask. Maybe they should start asking about mental health in MIPs, although support for a proposal does not necessarily mean that the issue is salient.*

I thank the reviewer for emphasizing the distinction between importance and salience, and for the suggestion to incorporate MIP data. I have added a paragraph on the distinction with a reference to recent Gallup data in lines 384-396 of the tracked-changes revision. Here I draw on Moniz and Wlezien’s argument that importance is a necessary but not sufficient condition for salience, which seems to apply here (however important I found mental health to be in this experiment, it is clearly not salient in terms of open-ended responses to the MIP question).

*There is research on public support for mental health outside of the US. For example, a paper that uses data from England (Bernardi 2021a) shows that support for mental health services is very high among the English public. So, this evidence is not unique to the US and acknowledging this can strengthen the message of the paper.*
I thank the reviewer for this helpful suggestion – I have incorporated a reference to Bernardi’s findings in lines 86-89 of the tracked-changes revision. Additionally, in the following paragraph I have changed “Americans” to “people” (line 91).

*Mental health may be a valence issue (see Stokes 1992) – there is wide agreement on the goals at least, maybe not on the means –, that is, support may be high on mental health services and a relationship may be found with voting propensity.*

I have added a discussion of mental health as a valence issue with a citation to Stokes in lines 75-78 of the tracked-changes revision.

*However, a totally different matter – in the US – is support for a candidate with mental health problems. The available experimental research (Loewen and Rheault 2021; Magni and Reynolds 2024) tells us that candidates with mental health problems suffer an electoral penalty. I believe such discussion should be incorporated in the paper. Mental illness stigma still is relevant in the US.*

I have incorporated a reference to the issue of stigma toward candidates and a citation to Magni and Reynolds 2024 in lines 57-58 in the revised version. *The paper dwells between mental health as a societal problem and as a public opinion’s priority. But the author does not engage at all with any of this research. As it stands, the paper is entirely empirical with no attempt to include any theoretical discussion or to connect the findings to existing research. Why is there a positive effect of public approval of a mental health policy proposal (but also other proposals, based on Figure 1) and candidate support? For example, according to Kristensen et al. (2023: 2855), “If a problem indicator shows that a problem is severe, then parties across the board attend to the problem.” In other words, candidates who advance policy proposals about pressing problems may be rewarded electorally because they are paying attention to such problems. Alternatively, as Fournier et al. (2003) suggest, when asked to evaluate the government on a certain issue when it is important to them, people are more likely to connect their evaluation to their vote choice. (For a broader discussion on issue salience I recommend the entry by Moniz and Wlezien (2020).)*

While the literature review of this short article is necessarily brief, I agree with the reviewer that it would be strengthened with a clearer connection to existing theory and findings in political behavior. To that end, I have added text in lines 100-105 of the tracked-changes revision grounding my questions on importance and vote choice in this literature, with references to Krosnick 1988 and Fournier et al 2003. (As I noted above, I have also incorporated a reference to Moniz and Wlezien elsewhere in the manuscript.)

*And why is support for mental health so high in the US? Is it because mental health is a valence or quasi-valence issue (Bernardi 2021b)? In the UK, PhD research by Daniel Bowman (2025) shows that politicians’ attention to mental health has increased over time and the main parties (Conservative, Labour, Liberal Democrats) and parliamentarians from those parties all care about mental health to some extent but they frame the issue differently in election manifestos or parliamentary debates. Overall, those are just some examples of literature from issue salience / agenda setting, but it would be nice if this paper could be a bit more theory-inspired and could provide some discussion of the results in that direction. The author may not have pre-registered hypotheses, but he/she should attempt to explain what is going on in their data.*

See my responses above on the “valence issue” concept and references to the literature. *Why are results presented with 90% and not 95% confidence intervals?*
I made this decision because of the relatively low statistical power of the study (by the standards of conjoint experiments), but it makes little difference to the findings. In response to this question and a similar comment from Reviewer #3, I have changed both figures to display 95% confidence intervals and edited the text (including figure captions) to match. *Of course, the experiment has been conducted, but I still wonder about the treatment condition for mental health. It is hard to disagree with such a statement, especially if one is only given the possibility to support or oppose the policy, without any opportunity to use a degree of options. In this regard, the author has asked about strength of support/opposition. How do results look like when a variable that incorporates those options is used? I think the author can construct an ordinal variable from the two original variables."*

I thank the reviewer for the suggestion. I have added a new regression table to Appendix S4 (Table S4.6) that interacts the agreement variable with the respondent’s specific position on the mental health issue proposal, operationalized as a single dummy variable for strong vs. weak support or opposition (model 1) and as a series of dummies indicating the respondent’s choice among the four options (with “strong opposition” as the excluded category). The model 1 results suggest that the agreement effect on vote choice is much stronger for those with strongly held opinions, as expected. The model 2 results suggest this is driven by the 736 respondents who selected “strong support” rather than the mere 34 who selected “strong opposition,” though the latter group is too small to make definitive conclusions. Unfortunately, I cannot replicate this analysis for the other nine issues, since the Common Content items include only “support” and “oppose” as response options. I discuss the drawbacks of the dichotomous nature of the questions and reference the new analyses in lines 420-436 of the tracked-changes revision.

**Reviewer #2**

*Thank you for the opportunity to review this interesting and well-executed study. The manuscript presents a timely and valuable contribution by shifting attention from general attitudes toward mental health policy—where public support is already high—to the question of whether mental health actually influences vote choice. The experimental design using a conjoint approach embedded in the CES is methodologically appropriate and well justified, and the findings are clear: mental health policy positions meaningfully affect respondents’ vote choices at levels comparable to other prominent issues. This offers an important addition to the literature on issue priorities in public opinion.
The analysis is rigorous overall, and the write-up is strong. I have several suggestions that could strengthen the manuscript further:
1. Subgroup analyses — The discussion would benefit from slightly more explicit emphasis on the imprecision of effects for smaller demographic groups. This is acknowledged, but a bit more caution in interpretation within the Results section would improve clarity.*

I have added an acknowledgment of this issue to lines 338-342 in the tracked-changes revision, with a citation to Schuessler and Freitag’s paper on statistical power in conjoint analyses (including for conditional/subgroup AMCEs). I also added a citation to the same paper to my existing discussion of this issue in the conclusion (see lines 398-399 in the revision).

*2. Item-order effect — The detection of a significant preference for Candidate A and the analytic adjustment for this are important. Briefly elaborating on the theoretical or cognitive reasons such effects occur in conjoint tasks could help contextualize this issue for readers.*

I have added a sentence on the known issue of screen position effects in self-administered experiments, reflecting respondent use of an unconscious “up (or first) means good” heuristic, with a citation to Tourangeau, Couper and Conrad 2013 (see lines 275-280 in the track-changes revision).

*3. Generality of the mental health proposal — While the argument that voters respond to broad thematic categories is reasonable, a bit more discussion of how issue framing may influence relative salience would strengthen external validity claims.*

I thank the reviewer for this helpful suggestion. I have added a brief discussion of the framing issue (with a cite to Nelson and Oxley 1999) in lines 408-411 of the tracked-changes revision, and edited the following sentence to emphasize that Alvarez and Morrier use the method I employ in this study, which is distinct from Nelson and Oxley’s.

*4. Minor polish — There are a few formatting and typographical inconsistencies that can be easily corrected during revision (e.g., spacing and line break artifacts from the draft format).*

I apologize for these errors and inconsistencies, and I hope I have rectified them in the revised submission.

*Despite these modest suggestions, the study meets the technical and reporting criteria for PLOS ONE and adds meaningful evidence that mental health is not only symbolically supported but also politically consequential. I encourage the authors to continue developing this line of work.*

**Reviewer #3**

*Introduction:
Could you add more about the rationale? And a clear objective. Your objective has been spread all over the introduction. I understand what you intend to do, however, we need a clear and specific written objective.*

I have changed the opening (lines 43-44 of the tracked-changes revision) to establish the research question in the first sentence: “How important is mental health to the American public as a policy issue?”

*Comment: Data and Variables : you said you sampled 1000 randomly, but how many where there in total? And did you do the randomization? If you didn’t opt for any randomization technique, then you should probably say that you sampled them using convenience sampling. Otherwise, explicitly mention how you randomized them.*

The sample was not recruited by me, but by YouGov in collaboration with the Cooperative Election Study (CES) team leads. The CES uses sample matching which is a probability sampling method that does involve randomization: the “target sample” is randomly selected from American Community Survey microdata, and YouGov panelists are matched to those individuals using proximity matching (this is the “matched sample” that participates in the survey). I have expanded an explanation of the method in lines 171-177 of the tracked-changes manuscript, adding a reference to pages 14-19 of the cited CES Guide which provides a full explanation of the theory and procedure of sample matching as well as validation of its accuracy. I also clarify that the team module of 1,000 respondents used for this study is randomly selected by YouGov from the 60,000-respondent Common Content sample recruited by YouGov.

*Comment: in line 163-165, you said “implement the conjoint experiment using a combination of 164 original questions and items from the CES Common Content 165 questionnaire.”. Since you are referring to something, putting a reference would be a better choice.*

I have added a reference to the CES Guide, which includes the Common Content questionnaire (line 184 in the tracked-changes revision).

*Comment: In line 209-211, you said, “I re-code the randomized candidate issue positions to 210 indicate whether the candidate agrees with the respondent (1) or 211 not (0). ”. What do you mean by re-code? Clarify a little bit. Did you mean post-coding?*

I apologize for the confusion caused by my imprecise use of “re-code,” which implied that I was changing an existing variable in some way. I have revised this passage to make clear that I *created* dichotomous variables for each randomly generated candidate issue position indicating whether or not the respondent agreed with the candidate’s position (lines 228-230) in the tracked-changes revision). I believe this is more accurate and less confusing than the phrasing in the initial submission.

*Comment: In line 211-212, you said “These agreement variables are the key independent 212 variables (see “Analysis” for more on methodology)”, here you are referring to something you described later. Usually reference is made to something described earlier. However, a little more is required to understand the concept of “agreement variables”. You didn’t mention this terminology earlier.*

I believe this confusion also followed from the imprecise phrasing referenced in the previous comment. I have also edited this sentence to refer to “These indicators of respondent-candidate agreement” (line 231 of the tracked-changes revision) to make clear that I am referring to the variables I described in the previous sentence. I have also cut the parenthetical reference to the “Analysis” section in that sentence.

*Comment: Then in line 213-216, you said “For the heterogeneous effects analysis, I make use of the 214 Common Content’s battery of demographic and political questions, 215 collapsing some categories to achieve adequate subgroup sample 216 sizes.”. Reference is needed here to justify that whatever you are doing scientifically appropriate.*

I have edited this sentence and added a citation to Schuessler and Freitag 2020 in lines 237-238 of the tracked-changes revision to emphasize the importance of subgroup size for heterogeneous effects analysis in a conjoint experiment. Also see my responses to other comments related to sample and subgroup size.

*Comment: The authors should indicate whether the study adhered to established ethical standards (e.g., the Declaration of Helsinki). Although the manuscript states that the study was approved by the Institutional Review Board (IRB), it would be helpful to explicitly mention compliance with relevant ethical guidelines. If the research complied with these standards, please state this clearly in the Ethics section.*

The revised text in lines 254-259 of the tracked changes revision makes clear that the study followed the established ethical standards of no more than minimal risk to participants and written informed consent.

*Comment: In line 236-237, you mentioned “I follow the analytical strategy of Alvarez and Morrier with a 237 slight modification. ”. Again you are referring to something, need to put a reference.*
I have added the reference (see line 262 in the tracked-changes revision).

*Comment: Do you have explicit reason behind using 90% CI. Usually 95% is used. If you are using 90%, you made concrete justification for that.*
I have switched to 95% confidence intervals in both figures and edited the text and table captions to match (also see Reviewer 1’s similar question above).

*Comment: In the discussion and conclusion you mention relatively small sample size as one of you limitations. However, 1000 participant is not a small sample.*

I believe the changes I made in response to Reviewer 2’s comment on the imprecision of effects for subgroup analyses (see above) have addressed this question: the effective sample sizes of 2,320 to 2,452 respondent-profile observations (see lines 224-228 in the tracked-changes revision) are small in the context of conjoint experiments, where effective sample sizes in the 10,000s are the norm (see Schuessler and Freitag 2020, which I now cite in lines 238, 341 and 399 in the tracked-changes revision). This is because conventional conjoint experiments use all respondent-profile observations, while this design uses only those observations in which the policy issue in question appears (again, see lines 224-228 for an explanation). So, the key limitation is not that I have only 1,000 respondents (a respectable number), but that I am not able to use all 12,000 respondent-profile observations. This is not an issue for distinguishing the very large main ACMEs from 0 (as in Figure 1), but it does limit my ability to detect the much smaller differences *between* those estimates (if any), and to detect subgroup effects as in Figure 2 (an issue Schuessler and Freitag also address).

*Comment: Appendix S2. The frequency table is not representative. If you could add percentage along with the frequency, it would better represent.*

This table (S2.1) does display percentages calculated with sampling weights. The version in the initial submission may have caused confusion because of the title (“Frequency distributions…”) and the lack of percentage symbols in the table. I have changed “Frequency distributions” to “Distributions” and added percentage symbols. I have also converted the proportions in Table S2.2 to percentages for consistency.

*Comment: Appendix S4 is a bit confusing. Try to incorporate more information about the items of table in the suffix.*

I have added text to the note of each table in Appendix S4 referring readers to the descriptive statistics in Appendix S2 for information on these variables.

**Additional changes**

Lastly, in my own review of the original submission, I noticed several small typographical errors in Appendices S3 and S4, which I have corrected in the revised version.

Again, I thank all of you for taking the time to read my manuscript and make such constructive and helpful suggestions.

Best,

Jake Haselswerdt, PhD

Truman School of Government & Public Affairs

University of Missouri
